# Supplementary material for: Endocardial Fibroelastosis as an Independent Predictor of Atrioventricular Valve Rupture in Maternal Autoimmune Antibody Exposed Fetus: A Systematic Review with Clinicopathologic Analysis
Source: Diagnostics (Basel). 2023 Apr 20;13(8):1481. doi: 10.3390/diagnostics13081481 (PMC10137427; doi:10.3390/diagnostics13081481)
Supplement: Supplementary file 1 [file diagnostics-13-01481-s001.zip › Supplementary material/Supplementary material.pdf]

Supplementary tables:

Table 1: PRISMA checklist

Section/topic	#	PRISMA Checklist item	Reported on page #
**TITLE**			
Title	1	Identify the report as a systematic review, meta-analysis, or both.	1
**ABSTRACT**			
Structured summary	2	Provide a structured summary including, as applicable: background; objectives; data sources; study eligibility criteria, participants, and interventions; study appraisal and synthesis methods; results; limitations; conclusions and implications of key findings; systematic review registration number.	1-2
**INTRODUCTION**			
Rationale	3	Describe the rationale for the review in the context of what is already known.	3
Objectives	4	Provide an explicit statement of questions being addressed with reference to participants, interventions, comparisons, outcomes, and study design (PICOS).	3
**METHODS**			
Protocol and registration	5	Indicate if a review protocol exists, if and where it can be accessed (e.g., Web address), and, if available, provide registration information including registration number.	11
Eligibility criteria	6	Specify study characteristics (e.g., PICOS, length of follow-up) and report characteristics (e.g., years considered, language, publication status) used as criteria for eligibility, giving rationale.	11
Information sources	7	Describe all information sources (e.g., databases with dates of coverage, contact with study authors to identify additional studies) in the search and date last searched.	11
Search	8	Present full electronic search strategy for at least one database, including any limits used, such that it could be repeated.	Supplement- Search stratergy report
Study selection	9	State the process for selecting studies (i.e., screening, eligibility, included in systematic review, and, if applicable, included in the meta-analysis).	11
Data collection process	10	Describe method of data extraction from reports (e.g., piloted forms, independently, in duplicate) and any processes for obtaining and confirming data from investigators.	11
Data items	11	List and define all variables for which data were sought (e.g., PICOS, funding sources) and any assumptions and simplifications made.	11
Risk of bias in individual studies	12	Describe methods used for assessing risk of bias of individual studies (including specification of whether this was done at the study or outcome level), and how this information is to be used in any data synthesis.	11
Summary	13	State the principal summary measures (e.g., risk ratio, difference in	N/A

measures		means).	
Synthesis of results	14	Describe the methods of handling data and combining results of studies, if done, including measures of consistency (e.g., I^2) for each meta-analysis.	N/A
**RESULTS**			
Study selection	17	Give numbers of studies screened, assessed for eligibility, and included in the review, with reasons for exclusions at each stage, ideally with a flow diagram.	Figure 9
Study characteristics	18	For each study, present characteristics for which data were extracted (e.g., study size, PICOS, follow-up period) and provide the citations.	Table 1,2.3
Risk of bias within studies	19	Present data on risk of bias of each study and, if available, any outcome level assessment (see item 12).	Supplement table 2
Results of individual studies	20	For all outcomes considered (benefits or harms), present, for each study: (a) simple summary data for each intervention group (b) effect estimates and confidence intervals, ideally with a forest plot.	N/A
Synthesis of results	21	Present results of each meta-analysis done, including confidence intervals and measures of consistency.	N/A
Risk of bias across studies	22	Present results of any assessment of risk of bias across studies (see Item 15).	N/A
Additional analysis	23	Give results of additional analyses, if done (e.g., sensitivity or subgroup analyses, meta-regression [see Item 16]).	N/A
**DISCUSSION**			
Summary of evidence	24	Summarize the main findings including the strength of evidence for each main outcome; consider their relevance to key groups (e.g., healthcare providers, users, and policy makers).	21-25
Limitations	25	Discuss limitations at study and outcome level (e.g., risk of bias), and at review-level (e.g., incomplete retrieval of identified research, reporting bias).	25-26
Conclusions	26	Provide a general interpretation of the results in the context of other evidence, and implications for future research.	26
**FUNDING**			
Funding	27	Describe sources of funding for the systematic review and other support (e.g., supply of data); role of funders for the systematic review.	N/A

Search Strategy Report
Researcher: Kotecha Monika Kantilal
Topic: Atrioventricular valve rupture in neonatal lupus
Date: 15/8/2022
Update: 18/3/2023

PubMed

Set	Terms
#1	(Mitral OR tricuspid OR atrioventricular OR valve OR cardiac OR heart OR chord* OR valv* OR papillary) AND (rupture* OR insufficiency OR regurgi* OR repair) AND (humans[Filter])

#2	("Neonatal lupus" OR "maternal Lupus" OR "Maternal Autoimmunity" OR "maternal antibodies" OR lupus OR "Anti Ro" OR "anti La" OR "anti SSA" OR "anti SSB") AND (humans[Filter])
#3	#1 AND #2
#4	Echocardiography AND (humans[Filter])
#5	Autopsy OR Immunohistochemistry OR immunopathology AND (humans[Filter])
#6	#2 AND #4
#7	#2 AND #5

Embase

Set	Terms
#1	('rupture'/exp OR 'rupture') AND ('heart rupture':ti,ab,kw OR 'chordal rupture':ti,ab,kw OR 'mitral valve regurgitation':ti,ab,kw OR 'atrioventricular valve':ti,ab,kw OR 'tricuspid valve disease':ti,ab,kw OR 'tricuspid valve regurgitation':ti,ab,kw OR 'myopericarditis':ti,ab,kw OR 'lymphocytic myocarditis':ti,ab,kw OR 'myocarditis':ti,ab,kw OR 'papillary muscle rupture':ti,ab,kw OR 'heart valve surgery':ti,ab,kw OR 'mitral valve surgery':ti,ab,kw)
#2	'lupus' OR 'lupus erythematosus':ti,ab,kw OR 'autoimmunity':ti,ab,kw OR 'maternal antibody':ti,ab,kw OR 'antinuclear antibody':ti,ab,kw OR 'la antibody':ti,ab,kw OR 'lupus anticoagulant':ti,ab,kw OR 'ro 52 antibody':ti,ab,kw OR 'ro 60 antibody':ti,ab,kw OR 'ro antibody':ti,ab,kw
#3	#1 AND #2AND ([embryo]/lim OR [fetus]/lim OR [newborn]/lim OR [infant]/lim OR [child]/lim OR [preschool]/lim OR [school]/lim OR [adolescent]/lim)
#4	'endocardial fibroelastosis'/exp
#5	'autopsy'/exp
#6	'immunohistochemistry'/exp
#7	#2 AND #4 ([embryo]/lim OR [fetus]/lim OR [newborn]/lim OR [infant]/lim OR [child]/lim OR [preschool]/lim OR [school]/lim OR [adolescent]/lim)
#8	#2 AND #5 ([embryo]/lim OR [fetus]/lim OR [newborn]/lim OR [infant]/lim OR [child]/lim OR [preschool]/lim OR [school]/lim OR [adolescent]/lim)
#9	#2 AND #6 ([embryo]/lim OR [fetus]/lim OR [newborn]/lim OR [infant]/lim OR [child]/lim OR [preschool]/lim OR [school]/lim OR [adolescent]/lim)
#10	

Scopus:

Scopus query final:

(TITLE-ABS-KEY(("heart disease") OR heart OR mitral OR tricuspid OR chord* OR atrioventricular OR ("papillary muscle") OR valv* OR cardiac OR heart OR papillary AND (rupture* OR regurgitation* OR insufficienc* OR repair))) AND ("Neonatal lupus" OR "maternal Lupus" OR "Maternal Autoimmunity" OR "maternal antibodies" OR "Anti Ro" OR "anti La" OR "anti SSA" OR "anti SSB" OR lupus OR "Ro 52 antibody" OR "Ro 60 antibody" OR "Ro antibody") AND (LIMIT-TO (SUBJAREA,"MEDI") OR LIMIT-TO (SUBJAREA,"BIOC") OR LIMIT-TO (SUBJAREA,"IMMU") OR LIMIT-TO (SUBJAREA,"PHAR") OR LIMIT-TO (SUBJAREA,"HEAL") OR LIMIT-TO (SUBJAREA,"MATE") OR LIMIT-TO (SUBJAREA,"DECI") OR LIMIT-TO (SUBJAREA,"Undefined") OR LIMIT-TO (SUBJAREA,"MULT")) AND (EXCLUDE (EXACTKEYWORD,"Middle Aged") OR EXCLUDE (EXACTKEYWORD,"Nonhuman") OR EXCLUDE (EXACTKEYWORD,"Aged, 80 And Over") OR EXCLUDE (EXACTKEYWORD,"Animals") OR EXCLUDE (EXACTKEYWORD,"Middle Age") OR EXCLUDE (EXACTKEYWORD,"Very Elderly") OR EXCLUDE (EXACTKEYWORD,"Animal") OR EXCLUDE (EXACTKEYWORD,"Kidney Failure") OR EXCLUDE (EXACTKEYWORD,"Renal Insufficiency, Chronic") OR EXCLUDE (EXACTKEYWORD,"Acetylsalicylic Acid") OR EXCLUDE (EXACTKEYWORD,"Animal Experiment") OR EXCLUDE (EXACTKEYWORD,"Mouse") OR EXCLUDE (EXACTKEYWORD,"Mice") OR EXCLUDE (EXACTKEYWORD,"Cyclophosphamide") OR EXCLUDE (EXACTKEYWORD,"Animal Tissue") OR

EXCLUDE (EXACTKEYWORD,"Young Adult") OR EXCLUDE (EXACTKEYWORD,"In Vitro Study") OR EXCLUDE (EXACTKEYWORD,"Kidney")) AND (EXCLUDE (EXACTKEYWORD,"Adult") OR EXCLUDE (EXACTKEYWORD,"Deep Vein Thrombosis"))

Web of science:

Query 1: **TS=(("heart disease") OR heart OR mitral OR tricuspid OR chord* OR atrioventricular OR papillary OR valv* OR cardiac OR heart OR papillary AND (rupture* OR regurgitation* OR insufficienc* OR repair))** and **1.37 Cardiology - General** or **1.94 Cardiac Arrhythmia** or **1.71 Cardiology - Circulation** or **1.218 Autonomic Regulation** or **1.79 Molecular & Cell Biology - Physiology** or **1.134 Trauma & Emergency Surgery** or **1.154 Assisted Ventilation** or **1.102 Stem Cell Research** or **1.75 Blood Clotting** or **1.72 Obstetrics & Gynecology** or **1.168 Vascular, Cardiac & Thoracic Surgery** or **1.6 Immunology** or **1.159 Membrane Channels & Receptors** or **1.25 Molecular & Cell Biology - Cancer, Autophagy & Apoptosis** or **1.96 Cell Biology** or **1.106 Rheumatology** or **1.132 Extracellular Matrix & Cell Differentiation** or **7.57 Modelling & Simulation** or **1.208 Vasculitis & Autoimmune Disorders** or **1.281 Auto-inflammatory Diseases** or **1.156 Healthcare Policy** or **1.189 Genome Studies** or **1.257 Birth Defects** or **1.175 Medical Physics** or **1.155 Medical Ethics** or **1.204 Molecular & Cell Biology - Immunotherapy** or **1.103 Blood Disorders** or **2.123 Protein Stucture, Folding & Modelling** or **1.286 Immunology & Hematology** or **8.212 Sensors & Tomography** or **5.250 Imaging & Tomography** or **6.11 Education & Educational Research** or **1.152 Molecular & Cell Biology - Dna Damage** (Citation Topics Meso) and **Cardiac Cardiovascular Systems** or **Surgery** or **Peripheral Vascular Disease** or **Medicine General Internal** or **Pharmacology Pharmacy** or **Physics Applied** or **Anatomy Morphology** or **Critical Care Medicine** or **Biochemistry Molecular Biology** or **Physiology** or **Cell Biology** or **Medicine Research Experimental** or **Pediatrics** or **Hematology** or **Emergency Medicine** or **Multidisciplinary Sciences** or **Obstetrics Gynecology** or **Immunology** or **Health Care Sciences Services** or **Rheumatology** or **Medical Laboratory Technology** or **Medical Informatics** or **Microscopy** or **Allergy** or **Ethnic Studies** or **Education Educational Research** or **Primary Health Care** (Web of Science Categories) and **Cardiovascular System Cardiology** or **General Internal Medicine** or **Surgery** or **Pharmacology Pharmacy** or **Physiology** or **Biochemistry Molecular Biology** or **Cell Biology** or **Research Experimental Medicine** or **Pediatrics** or **Hematology** or **Science Technology Other Topics** or **Radiology Nuclear Medicine Medical Imaging** or **Rheumatology** or **Biophysics** or **Immunology** or **Medical Laboratory Technology** or **Medical Informatics** or **Pathology** or **Biotechnology Applied Microbiology** or **Anatomy Morphology** or **Materials Science** or **Allergy** or **Imaging Science Photographic Technology** or **Medical Ethics** (Research Areas)

Query 2: **TS=("Neonatal lupus" OR "maternal Lupus" OR "Maternal Autoimmunity" OR "maternal antibodies" OR "Anti Ro" OR "anti La" OR "anti SSA" OR "anti SSB" OR lupus OR "Ro 52 antibody" OR "Ro 60 antibody" OR "Ro antibody")** and **1.106 Rheumatology** or **1.6 Immunology** or **1.75 Blood Clotting** or **1.208 Vasculitis & Autoimmune Disorders** or **1.286 Immunology & Hematology** or **1.25 Molecular & Cell Biology - Cancer, Autophagy & Apoptosis** or **1.72 Obstetrics & Gynecology** or **1.103 Blood Disorders** or **1.37 Cardiology - General** or **1.54 Molecular & Cell Biology - Genetics** or **1.239 Tissue Barriers** or **1.65 Allergy** or **1.102 Stem Cell Research** or **1.132 Extracellular Matrix & Cell Differentiation** or **1.127 Molecular & Cell Biology - Pharmacology** or **1.141 Hormone Therapy** or **1.189 Genome Studies** or **1.281 Auto-inflammatory Diseases** or **1.204 Molecular & Cell Biology - Immunotherapy** or **1.94 Cardiac Arrhythmia** or **1.71 Cardiology - Circulation** or **1.257 Birth Defects** or **1.112 Palliative Care** or **9.92 Statistical Methods** or **1.168 Vascular, Cardiac & Thoracic Surgery** or **1.155 Medical Ethics** or **1.96 Cell Biology** or **1.225 Hematologic Diseases** or **1.307 Laboratory Medicine** or **5.250 Imaging & Tomography** or **1.254 Ultrasound In Medicine** or **6.3 Management** (Citation Topics Meso) and **Rheumatology** or **Immunology** or **General Internal Medicine** or **Hematology** or **Research Experimental Medicine** or **Biochemistry Molecular Biology** or **Cardiovascular System Cardiology** or **Cell**

Biology or **Pathology** or **Science Technology Other Topics** or **Pediatrics** or **Genetics Heredity** or **Obstetrics Gynecology** or **Oncology** or **Medical Laboratory Technology** or **Allergy** or **Surgery** or **Radiology Nuclear Medicine Medical Imaging** or **Transplantation** or **Biotechnology Applied Microbiology** or **Life Sciences Biomedicine Other Topics** or **Medical Informatics** or **Anatomy Morphology** or **Microscopy** (Research Areas)

Final query

#1 AND #2 and **Rheumatology** or **Cardiac Cardiovascular Systems** or **Medicine General Internal** or **Immunology** or **Pediatrics** or **Peripheral Vascular Disease** or **Medicine Research Experimental** or **Surgery** or **Hematology** or **Biochemistry Molecular Biology** or **Critical Care Medicine** or **Obstetrics Gynecology** or **Radiology Nuclear Medicine Medical Imaging** or **Multidisciplinary Sciences** or **Cell Biology** or **Allergy** or **Pathology** or **Pharmacology Pharmacy** or **Medical Laboratory Technology** or **Transplantation** or **Health Care Sciences Services** or **Physiology** or **Primary Health Care** (Web of Science Categories) and **Rheumatology** or **Cardiovascular System Cardiology** or **General Internal Medicine** or **Immunology** or **Pediatrics** or **Research Experimental Medicine** or **Respiratory System** or **Surgery** or **Hematology** or **Biochemistry Molecular Biology** or **Cell Biology** or **Obstetrics Gynecology** or **Radiology Nuclear Medicine Medical Imaging** or **Science Technology Other Topics** or **Allergy** or **Pathology** or **Pharmacology Pharmacy** or **Medical Laboratory Technology** or **Reproductive Biology** or **Biotechnology Applied Microbiology** or **Emergency Medicine** or **Anatomy Morphology** or **Medical Ethics** or **Health Care Sciences Services** or **Life Sciences Biomedicine Other Topics** or **Physiology** or **Social Issues** (Research Areas)

Table 2: JBI Quality assessment tool

	Cuneo et al 2011[8]	Cuneo et al 2009[9]	Weber et al 1994[10]	Shiraishi et al 2014[11]*	Hamaoka et al 2009[12]	Bellon et al 2022[13]	Tarca et al 2017[14]	Gonzalez-Lopez et al 2017[15]	Brooks et al 2015[16]	Fleming et al 2008[17]	Mawad al 2020[6]#
1. Were patient's demographic characteristics clearly described?	Yes	Yes	Yes	No	Yes	Yes	Yes	Yes	Yes	Yes	no
2. Was the patient's history clearly described and presented as a timeline?	Yes	Yes	Yes	No	Yes	Yes	Yes	Yes	Yes	Yes	no
3. Was the current clinical condition of the patient on presentation clearly described?	Yes	Yes	Yes	Yes	Yes	Yes	Yes	Yes	Yes	Yes	no
4. Were diagnostic tests or assessment methods and the results clearly described?	Yes	Yes	Yes	Yes	Yes	Yes	Yes	Yes	Yes	Yes	yes

5. Was the intervention(s) or treatment procedure(s) clearly described?	Yes	Yes	Yes	Unclear	Yes	Yes	Yes	Yes	Yes	Yes	no
6. Was the post-intervention clinical condition clearly described?	Yes	Yes	Yes	Unclear	Yes	Yes	Yes	Yes	Yes	Yes	no
7. Were adverse events (harms) or unanticipated events identified and described?	Yes	Yes	Yes	Unclear	Yes	Yes	Yes	Yes	Yes	Yes	no
8. Does the case report provide takeaway lessons?	Yes	Yes	Yes	Yes	Yes	Yes	Yes	Yes	Yes	Yes	yes

Low bias = 6 yes; Moderate bias = 4 yes; High bias = 2 or less yes).
*Shiraishi et al is survey of 94 cases of mitral valve rupture of which we selected only one case with neonatal lupus at etiology and hence we use JBI tool for this case.
Mawad et al is a research article on effect of steroids on lupus positive pregnancy. Atrioventricular valve rupture is noted in two patients however due to high bias the study was excluded.
